# Crushing injuries of the foot and ankle, with complex open fractures: result of a prospective study with a 3 year follow-up

**Published:** 2016

**Authors:** DA Edelstein, I Florescu

**Affiliations:** * “Bagdasar Arseni” Clinical Emergency Hospital, Bucharest, Romania; ** Floreasca Clinical Emergency Hospital, Bucharest, Romania

**Keywords:** crushing injuries, foot, ankle, open fractures, limb reconstruction

## Abstract

The objective of this study was to determine the compared results of both the reconstruction surgery and the amputation in severe crushing of the foot, which led to open fractures.

**The type of study.** Prospective.

**Background.** Two major trauma hospitals (Floreasca Clinical Emergency Hospital and “Bagdasar Arseni” Clinical Emergency Hospital) from the university center in Bucharest.

**Patients.** 21 patients, who sustained crushing of the foot with resulting Gustilo type III open fractures, were involved. The exclusion criteria were represented by open fractures that had very gross destructions of the neurovascular bundle, for which the amputation was the only solution, with no modality to reconstruct whatsoever.

**Treatment.** An immediate amputation (at 24, 48 hours after a thorough debridement, proper patient resuscitation, and detailed imaging investigation – the technique of delayed emergency) and reconstruction surgery were performed.

**Methods of evaluation.** Three variables were used: the Sickness Impact Profile (SIP) score, the Visual Analogue Scale (VAS) for the residual pain and the number of rehospitalizations for secondary surgical procedures.

**Results.** When comparing the two lots of patients, first in which the amputation patients were included and second in which the reconstruction patients were included, it was noticed that there was a less favorable prognostic in the second lot for a three-year follow up period.

**Conclusions.** The patients with a mangled foot, in which reconstruction surgery of the bone and soft tissue envelope was performed, had a worse prognostic than those who had an amputation as a first intention.

**Abbreviations**:

SIP = Sickness Impact Profile, VAS = Visual Analogue Scale, MVA = Motor Vehicle Accident, STSG = Split Thickness Skin Graft

## General presentation

Injuries of the foot and ankle are the result of high intensity forces, most frequently after MVA, construction site accidents and accidents in the rural areas resulting from contact with farming equipment. Because of this, they represent a real challenge for the trauma surgeon and for the plastic surgeon. Limb salvage methods often require arthrodesis, extensive bone reconstructions, most of them needing bone grafting and soft tissue flaps (free or pedunculated) or STSG. Although plastic reconstruction techniques have come a long way, there are still a lot of unresolved problems at the reconstructed limb. Such problems are bulky flaps, which are perceived as unaesthetic and they often lack sensibility, which requires other surgeries. Because of this, these patients require custom fitted shoewear or special ambulation devices. Another problem is represented by the fact that the foot is a weight bearing structure so the reconstruction methods must address this problem as well. Considering all these, some authors concluded that in carefully selected cases, amputation is a viable solution to reconstruction.

The purpose of this study was to analyze a lot of patients with such lesions and to see if the reconstruction methods have a less favorable prognostic. The hypothesis of the study was that amputation has a better long term result [**1**,**2**,**4**].

## Patients and methods

This was a prospective study, ran in two major trauma hospitals, Floreasca Clinical Emergency Hospital and “Bagdasar Arseni” Clinical Emergency Hospital, from the university center Bucharest, with a follow up of 21 patients included, up to three years. The eligibility of the population in the study was made up of 21 cases of severe crushing of the foot, resulting in open fractures Gustilo III, five of which had extensions in the ankle as well. A number of five patients (23,8%) had their feet amputated as a first intention in the first 24-48 hours, after a proper resuscitation and detailed imaging studies. The decision was based on the advice of the main treating surgeon and his experience with that kind of injuries. Reconstruction surgery was decided for the rest of 16 patients (76,2%), after the initial 24-48 hours follow up [**3**,**6**,**7**]. Prospective dates were obtained from the patients through a clinical exam at 12, 24 and 36 months period. In both trauma centers, there was a consultant orthopaedic and trauma doctor with his team, who managed the initial treatment plan, and a consultant plastic surgery doctor with his team, who addressed the soft tissue defect. The plastic surgery team was consulted for the admission of the patient. After the initial hospital leave and for each of the following presentations, both teams were involved in the examination, to see the present state and the evolution of the patient. All the lesions were prospectively documented by using classic trauma classifications. These classifications were used to describe the bone lesion, the soft tissue lesion, and the neurovascular involvement.

**Chart 1:**
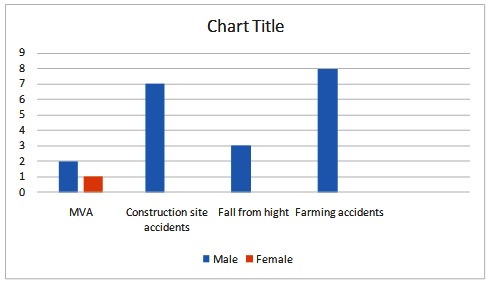
M: F ratio and leading cause distribution

Out of the 21 patients, only one was a woman (4,76%), the rest being men (95,2%). The leading causes of their injuries were as it follows: 3 MVA (14,28%), 7 construction site accidents (33,2%), 4 falls from height (19,04%) and 8 farming accidents (38,1%) (**Chart 1**). 

Out of the 16 patients with reconstruction surgeries, 14 of them (87,5%) required at least a second readmission for complications associated or for continuing the initial treatment. Out of them, 8 patients (50%) were readmitted for profound infections, 3 (18,75%) had no or vicious bone consolidation and another 3 patients (18,75%) developed secondary arthrosis which required arthrodesis.

Out of the five cases of amputated feet, only one patient (20%) was readmitted for an infected wound dehiscence, which required another amputation at the superior level.

**Evaluation methods**

For the evaluation of their general physio-psycho-social evolution, the following three instruments were used: the SIP score, which contained 80 questions from 10 different domains. These domains were walking, mobility, hygiene, social interaction, emotional behavior, rest, recreation, home activity, work activity. The higher the SIP score was, the higher the disability was. This score was used to evaluate the degree of functional evolution. Comparative calculi were made for all the 10 groups of evaluation [**2**,**10**].

The second score used in the patients was the VAS scale for the residual pain. This score subjectively evaluated the level of pain, which was indicated by the patient on a scale from one to ten.

The third evaluation factor of the psychosocial evolution of the patients was represented by the number of later readmissions for secondary surgical interventions.

At the proposed intervals (1, 2 and 3 years), patients were anamnestic and clinically examined by the teams of treating physicians. In this way, the rehabilitation of the affected limb was evaluated regarding the bone healing, soft tissue healing, secondary complications, and the necessity of reinterventions to address these complications.

The functionality of the limb was evaluated in comparison with the range of motion of the contralateral joints, regarding the residual pain and from the point of view of walking without aid of special devices and, in conclusion, the overall reintegration in society. The purpose of this analysis was to compare the results obtained in the patients with reconstruction surgery, versus those with amputation. The differences between the two groups were statistically compared.

## Results

The results obtained at the proposed intervals are outlined in **Table 1**-**3**. At the 12 months interval, 100% from the total of 21 patients returned for reevaluation, at 24 months 95,23% returned for reevaluation and at the 36 months interval only 80,9% of the patients initially included in the study returned for the reevaluation. Out of the reconstruction lot 87,5% of them needed at least one secondary surgical intervention, but from the amputation lot, only 20% needed a second intervention [**5**,**8**,**10**].

With this data available, we started from the hypothesis that the patients suited for the reconstruction procedures would have a higher SIP score and in the end a less favorable prognostic than the amputation patients. As a result, we noticed clinical and statistical differences between the two groups regarding the SIP score (p = 0.033).

At the same time, the number of readmissions for secondary surgical interventions, either for complications or staged procedures, at 1, 2 and 3 years interval was much higher in the reconstruction lot. This was statistically significant (p = 0.038).

**Table 1:** Patients follow-up at 12 months interval

Number of patients	Mean SIP score	Mean VAS score	Number of readmissions
5	11,6 ± 14,3	4,5 ± 5	1
16	18,2 ± 21,1	6,2 ± 6,4	14

**Table 2:** Patients follow-up at 24 months interval

Number of patients	Mean SIP score	Mean VAS score	Number of readmissions
5	12 ± 14,9	3,7 ± 4,1	0
15	13,6 ± 20,2	6,0 ± 7,0	8

**Table 3:** Patients follow-up at 36 months interval

Number of patients	Mean SIP score	Mean VAS score	Number of readmissions
3	10,5 ± 12,7	2,1 ± 2,6	0
14	15,6 ± 18,1	5,9 ± 6,5	3

## Discussions and conclusions

The role of this study was to analyze two lots of patients treated for Gustilo grade III open fractures of the foot, either by reconstruction surgery or by amputation. By some degree, it showed that patients with reconstruction of the bone and the soft tissue envelope had less favorable results than those with amputation as the primary treatment. There were also a higher number of readmissions, either planned as a part of the treatment, or as a result of late complications for the reconstruction patients. There was obviously no need for secondary planed surgeries for the amputee patients, only the reinterventions for the complications, which in this case, were very few, but also the number of patients with amputations was much lower than the ones with reconstruction. In the end, a longer time was needed for the reconstruction patients to achieve weight bearing, than those with amputation [**2**,**7**].

**Fig. 1:**
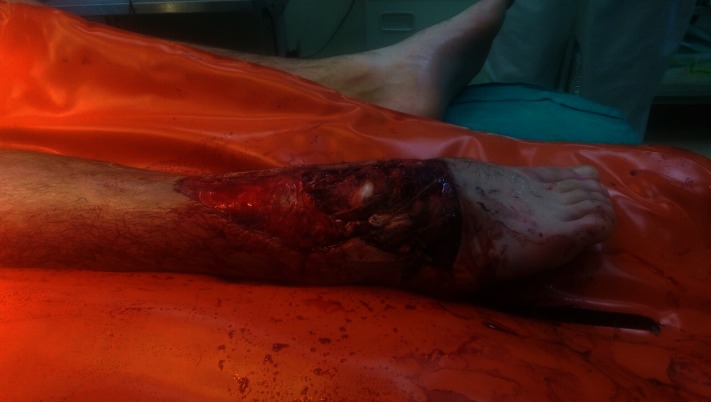
Open fracture of the ankle Gustilo IIIC, initial presentation, ER examination

**Fig. 2:**
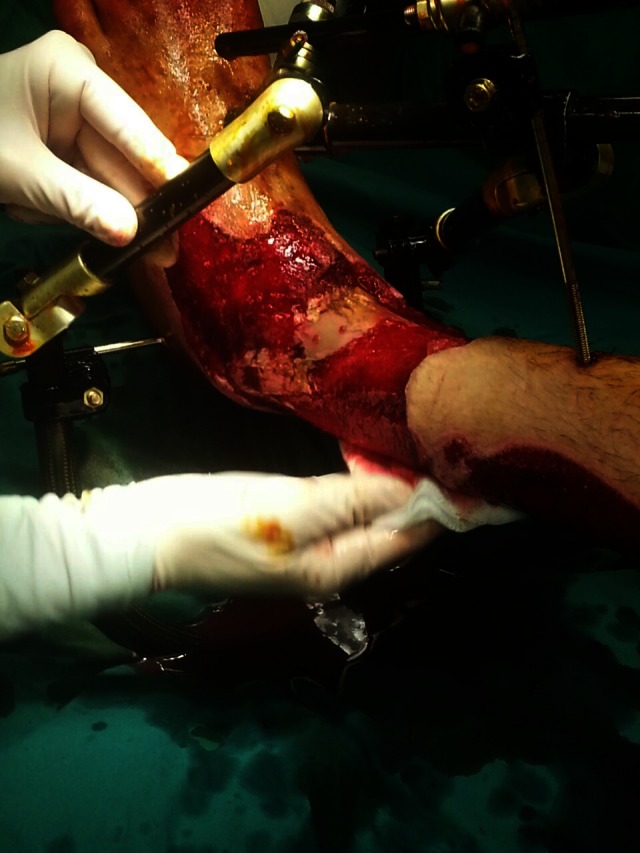
Open fracture of the ankle after initial debridement and external fixation

**Fig. 3:**
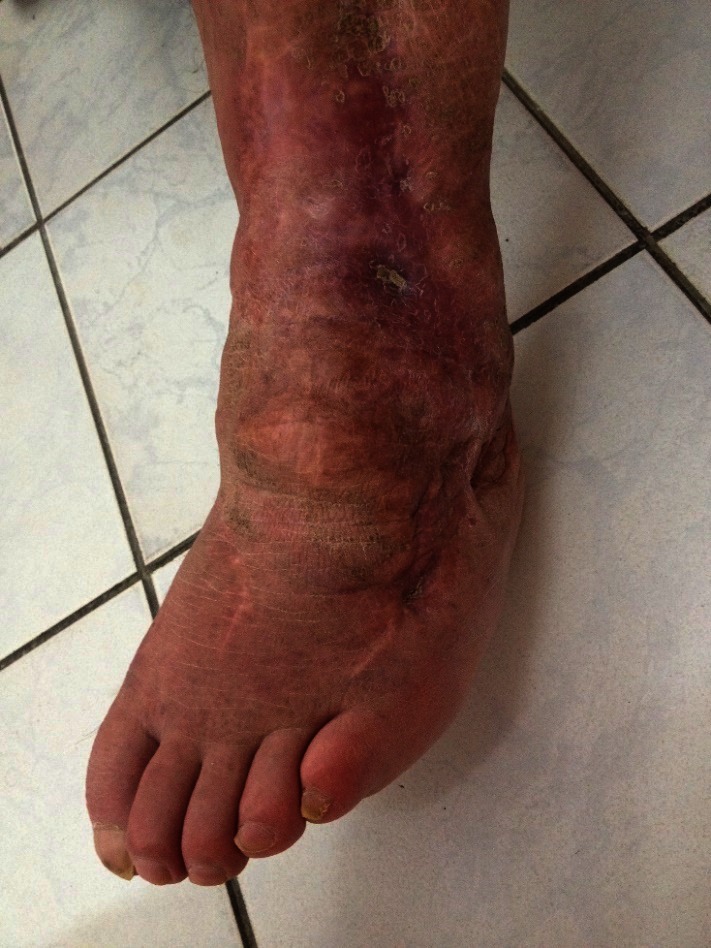
3-years follow up of an open ankle fracture Gustilo IIIB

This type of lesions are a real challenge for the treating physicians and has known issues after treatment like: loss of sensation in the plantar area, loss of the normal shape of the foot, which leads to the necessity of custom made shoes and walking aiding devices. As a morpho-functional unit, the foot sustains a huge weight pressure during stance and walking and this fact is very hard to reproduce after such massive injuries [**5**,**8**].

Studies from literature, like that of Sanders and collaborators and that of Ellington and collaborators, showed similar results of better outcomes on long term and a trend of the surgeons to treat Gustillo type IIIB and IIIC after crushing injuries, with primary amputation. Also, the LEAP study showed that in the case of reconstructive surgery following open fractures secondary to crushing trauma of the foot, there were unsatisfying long term results, mainly because the abnormal and unaesthetic shape of the foot affected shoe wear, comfort and personal performance. According to the LEAP study, another failure factor is the presence of secondary arthrosis and the need for arthrodesis [**9**,**10**].

However, this study could not show its efficiency because of the small number of patients in each lot. Also during the three-year follow up, a representative number of patients, specifically from the amputee lot failed to come back for reexamination.

In conclusion, patients with this type of injuries of the foot and ankle, who had the reconstruction surgeries for bone and soft tissues, had results inferior to the patients who had an amputation as a primary treatment method. At present, this data can only be used as an educational source for the patients and for correct information regarding the treatment methods. It is necessary that further studies are undergone on large lots of patients.
